# Clinical Cases in Medical Wards of Mercy Hospital

**Published:** 1871-06

**Authors:** 


### Clinical Reports.

### CLINICAL CASES IN MEDICAL WARDS OF MERCY HOSPITAL.

Clinic by PROF. DAVIS. From Notes by S.

MERCY HOSPITAL, *November 11, 1870.*

CASE I.—RHEUMATISM.—This man was taken sick last Monday. On admission, three days ago, he presented the following symptoms: Skin dry and husky; pulse 120; lips dry; face flushed and red; tongue very red, raw, and granular; much oppression of breathing without any evidence of cardiac or pulmonary trouble; wrists and hands swollen and painful, and some epigastric distress indicating an irritable condition of the alimentary canal, which I feared would lead to a regular typhoid condition.

With such an irritable condition of the mucous membrane, a strongly alkaline course of treatment would be very likely to induce vomiting and purging. Colchicum would also be found to operate very violently, producing bloody evacuations, etc. Wishing to restore the action of the skin and kidneys, and yet keep the bowels quiet, and also to lessen the fever and eliminate the morbid material which was causing the local disturbance, we ordered a powder consisting of pulv. Doveri, 6 grs., potass. nitras, 6 grs., and hydrarg. chlor. mite, 1 gr., to be given every four hours.

The Dover's powder we supposed to be sufficiently soothing to prevent the calomel from producing too much effect upon the alimentary canal.

To reduce the frequency of the pulse the following mixture was directed:

℞. Veratrum Viride ---------------------- ʒj.

Camp. Tinc. Opii. ------------------- ℥jss.

Spts. Nitre, Dulc. ------------------- ℥jss.

A teaspoonful to be given alternately with the powders.

He has now been on this treatment for 24 hours.

The flush has disappeared from the face and the surface is bathed with perspiration; pulse reduced in frequency to about 90, and is more natural; tongue is still red, but no undue action of the bowels, which have moved but once to-day; has also passed water once in the same time; his pains have abated, and the swelling is decidedly diminished. We would continue the same course of treatment unless it should produce too much of a sedative or nauseating effect, in which case the veratrum should be discontinued. The powders should be modified by the removal of the mild chloride to avoid any unnecessary influence of the mercurial, and if he has no evacuation for 24 hours should have a mild laxative; perhaps the best thing would be a tablespoonful of castor oil.

*Nov. 18th.*—The patient now seems to be entirely free from fever; skin cool; swelling entirely gone from joints; tongue much more natural. Would discontinue the fever mixture, and give the powders three times a day only. The patient will probably be up and about in a few days.

*January 31, 1871.*

CASE II.—ACUTE DYSENTERY.—This patient came into the hospital last Friday; was taken with acute dysentery the previous Wednesday. The attack commenced with slight chills and some general pains, more especially in the abdomen, where they were griping sharp and severe, with frequent desire to evacuate the bowels, and tenismus. Passages slimy and mixed with blood.

There was moderate fever, coated tongue, pulse slightly acclerated, and the temperature of the skin a little elevated. There are many remedies which have been used to arrest acute dysentery. Some physicians are in the habit of putting a patient in the first stage upon laxative salines till they obtain a free fecal evacuation, and continuing them for several days after.

I have never succeeded with the evacuant treatment, and so far as my observation goes, in most of the cases where it has appeared successful they have always been where enough of the salines had been given to evacuate the bowels, invariably combined them with opium, without which they would not have succeeded.

Another method is by the use of ipecac in large doses, given at once, and the earlier the better; 20 grs., for instance, at intervals of one to six hours, and this is claimed to be infallible. If the first dose vomits the second will generally be tolerated, it is said, and two to four doses are claimed to be sufficient to subdue the disease. I have faithfully and thoroughly tried it in but few instances. In about half of the cases it vomited effectually, no matter how many times I gave it. In some it produced the happiest effects; in others, where it could not be retained in the stomach, I have administered it successfully per enema of starch associated with laudanum.

This patient, before I saw him, had taken one dose of opium. Since then has been taking turpentine and laudanum emulsions, viz.:

℞. Ol. Terebinth ---- ------------------ ʒiij.

Tinct. Opium ---------------------- ʒiij.

Acacia ---------------------------- ʒiij.

Sacch. Alb. ----------------------- ʒiij.

Aqua Mentha. ----- ---------------- ℥iij.

M. One teaspoonful every four hours.

And a solution of carbolic acid, as follows:

℞. Carbolic Acid, Cryst.------------------6grs.

Tinct. Opii. Camp.------------------℥jss.

Glycerine ----------------------------℥ss.

Water ---------------------------------℥ij.

M. One teaspoonful every four hours alternately with the emulsion.

Improvement has been steady, and he is now almost entirely cured; has but one passage in 24 hours, and this nearly natural.

The method of treatment which I have usually followed after the first stage was past is to give, if the patient has not had free evacuations at the beginning of the attack, 4 or 5 grs. of calomel, either with or without 2 or 3 grs. of ipecac, followed in five or six hours by a laxative of castor oil, sulphate of magnesia, or Rochelle salts. As it is safe to assume in such cases that there is more or less fecal matter retained in the alimentary canal. Then putting them upon some combination that is sufficiently anodyne to overcome the pain, and to reduce the frequency of the discharges. Benzoin has some property that diminishes inflammatory action of the mucous membrane, especially after the first stage. It is not astringent or tonic, but it possesses an alterative influence that is valuable in the peculiar condition of the vessels that belongs to these cases after the acute stage.

This patient needs no additional treatment except to lessen the amount of medicine, and as the emulsion begins to nauseate and the passages occur but once in 24 hours, we may safely and profitably omit it.

*January 23, 1871.*

Case III.—Scirrhus at the Pyloric Orifice of the Stomach.—In this case we find a tumor of a hard unyielding feel, the most prominent point of which is at the umbillicus, and extending some distance above and below.

There is complete development of the cancerous cachexia, great emaciation, sallow hue of the skin, and some degree of œdema in the lower extremities, the result of an impoverishment of the blood.

The patient has been troubled with indigestion for several years, and within the last month has commenced to be annoyed with vomiting whenever food accumulates in the stomach. The food is then ejected with a little bile and thick ropy mucus. If she abstains from food and drink vomiting will not occur. The same result might be obtained by limiting the diet to sweet milk and lime water, in equal parts, taken every half or three-quarters of an hour. This amount would probably be absorbed without creating distress, but if a large quantity is taken it will produce increased muscular action in the walls of the stomach. The ingesta brought in contact with the pyloric orifice excites reflex action, and the food is expelled.

The most effectual method for securing relief in these cases is the restriction of the patient to a diet composed of bland simple substances capable of being absorbed by the coats of the stomach, and these should be given in small quantities so that what is taken at one time may be absorbed without leaving any accumulation to be carried through the pylorous.

In cases as far advanced as this, when the pyloric orifice has become sufficiently narrowed to embarrass the passage of food, the patients will frequently importune for something to move the bowels, but physic will only increase the distress. The bowels do not move, simply because there is nothing in them to excite peristaltic action, and the less they are interfered with the better.

When we find a case of this kind at any stage there is no reason in the experience of the past to suppose that the case will yield to treatment.

Iodine, carbolic acid, etc., have all been used without marked marked success in any instance.

The principal thing to be done is to regulate the diet, as before indicated, and put them upon such treatment as will soothe the pain and assist nutrition.

I have found more amelioration from the use of a solution of carbolic acid, rendered anodyne by paragoric, than from any other combination. The formula directed in this case was as follows:

℞. Carbolic Acid Crystals ................6grs.

Glycerine ................℥ss.

Tinct. Opii. Camp. ................℥jss.

Water ................℥ij.

M. Dose, one teaspoonful every three or four hours. This associated with the use of lime water and thin porridge occasionally in small quantities.

Have sometimes used a pill of conium and arsenite of sodium, but most generally if the stomach is the seat of the disease it has appeared to add to the distress, although in cancer of other organs, as the breast, uterus, etc., the combination has seemed to afford more relief than any other internal treatment.

